# Closed loop stimulation reduces the incidence of atrial high-rate episodes compared with conventional rate-adaptive pacing in patients with sinus node dysfunctions

**DOI:** 10.1093/europace/euae175

**Published:** 2024-06-28

**Authors:** Ennio C L Pisanò, Valeria Calvi, Miguel Viscusi, Antonio Rapacciuolo, Ludovico Lazzari, Luca Bontempi, Gemma Pelargonio, Giuseppe Arena, Vincenzo Caccavo, Chun-Chieh Wang, Béla Merkely, Lian-Yu Lin, Il-young Oh, Emanuele Bertaglia, Davide Saporito, Maurizio Menichelli, Antonino Nicosia, Domenico M Carretta, Aldo Coppolino, Chi Keong Ching, Álvaro Marco del Castillo, Xi Su, Martina Del Maestro, Daniele Giacopelli, Alessio Gargaro, Giovanni L Botto

**Affiliations:** Cardiology and Intensive Care Unit, Vito Fazzi Hospital, Lecce, Italy; Cardiology, G. Rodolico—San Marco University Hospital, Catania, Italy; Clinical and Interventional Arrhythmology, Sant’Anna e San Sebastiano Hospital, Caserta, Italy; Department of Advanced Biomedical Sciences, Federico II University of Naples, Naples, Italy; Clinical and Interventional Arrhythmology, Santa Maria Hospital, Terni, Italy; Cardiology, Bolognini Hospital, Seriate, Italy; Arrhythmology, Fondazione Policlinico Gemelli IRCCS Università Cattolica del Sacro Cuore, Roma, Italy; Cardiology and Intensive Care Unit, Apuane New Hospital, Massa, Italy; Cardiology and Intensive Care Unit, Miulli Regional Hospital, Acquaviva delle Fonti, Italy; Cardiology, Chang Gung Memorial Hospital—Linkou—CGMH, Taipei, Taiwan; Heart and Vascular Centre, Semmelweis University, Budapest, Hungary; Cardiology, National Taiwan University Hospital, Taipei, Taiwan; Cardiology, Seoul National University Bundang Hospital, Seongnam, Korea; Cardiology, Camposampiero Hospital, Padova, Italy; Cardiology, Infermi Hospital, Rimini, Italy; Cardiology, F. Spaziani Hospital, Frosinone, Italy; Cardiology, Giovanni Paolo II Hospital, Ragusa, Italy; Cardiology and Intensive Care Unit, Policlinico Consorziale, Bari, Italy; Cardiology and Intensive Care Unit, Elecrophysiology, SS Annunziata Hospital, Savigliano, Cuneo, Italy; Cardiology/Cardiovascular Surgery, National Heart Center, Singapore; Cardiology, Hospital Universitario 12 de Octubre, Madrid, Spain; Cardiology, Wuhan Asia Heart Hospital, Wuhan, China; Clinical Research Unit, BIOTRONIK Italia, Cologno Monzese, Milan, Italy; Clinical Research Unit, BIOTRONIK Italia, Cologno Monzese, Milan, Italy; Clinical Research Unit, BIOTRONIK Italia, Cologno Monzese, Milan, Italy; ASST Rhodense, Rho & Garbagnate Hospitals, Viale Carlo Forlanini, 95, 20024 Garbagnate Milanese, Milan, Italy

**Keywords:** Atrial high-rate episodes, Atrial fibrillation, Stroke, Rate-adaptive pacing, Closed loop stimulation, Accelerometer pacemaker sensor

## Abstract

**Aims:**

Subclinical atrial fibrillation (AF) is associated with increased risk of progression to clinical AF, stroke, and cardiovascular death. We hypothesized that in pacemaker patients requiring dual-chamber rate-adaptive (DDDR) pacing, closed loop stimulation (CLS) integrated into the circulatory control system through intra-cardiac impedance monitoring would reduce the occurrence of atrial high-rate episodes (AHREs) compared with conventional DDDR pacing.

**Methods and results:**

Patients with sinus node dysfunctions (SNDs) and an implanted pacemaker or defibrillator were randomly allocated to dual-chamber CLS (*n* = 612) or accelerometer-based DDDR pacing (*n* = 598) and followed for 3 years. The primary endpoint was time to the composite endpoint of the first AHRE lasting ≥6 min, stroke, or transient ischaemic attack (TIA). All AHREs were independently adjudicated using intra-cardiac electrograms. The incidence of the primary endpoint was lower in the CLS arm (50.6%) than in the DDDR arm (55.7%), primarily due to the reduction in AHREs lasting between 6 h and 7 days. Unadjusted site-stratified hazard ratio (HR) for CLS vs. DDDR was 0.84 [95% confidence interval (CI), 0.72–0.99; *P* = 0.035]. After adjusting for CHA_2_DS_2_-VASc score, the HR remained 0.84 (95% CI, 0.71–0.99; *P* = 0.033). In subgroup analyses of AHRE incidence, the incremental benefit of CLS was greatest in patients without atrioventricular block (HR, 0.77; *P* = 0.008) and in patients without AF history (HR, 0.73; *P* = 0.009). The contribution of stroke/TIA to the primary endpoint (1.3%) was low and not statistically different between study arms.

**Conclusion:**

Dual-chamber CLS in patients with SND is associated with a significantly lower AHRE incidence than conventional DDDR pacing.

## Graphical Abstract

**Graphical Abstract:**
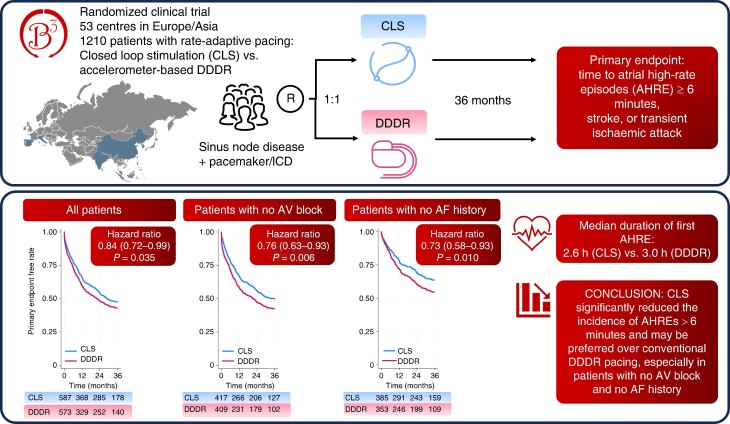

**What’s new?**

- In a multi-centre international trial, 1210 patients with sinus node dysfunctions and indication for dual-chamber pacing were 1:1 randomized to closed loop stimulation (CLS) (a rate-adaptive pacing driven by variations in ventricular contractility) or conventional accelerometer-based rate-adaptive (DDDR) pacing. The primary endpoint was the composite of first atrial high-rate episode (AHRE) ≥ 6 min, stroke, or transient ischaemic attack (TIA).
- During a 3-year follow-up, 53% of patients met the primary endpoint with a 16% relative risk reduction in the CLS vs. DDDR arm (*P* = 0.035; number needed to treat, 22.2), mostly driven by fewer AHREs lasting between 6 h and 7 days.
- The occurrence of strokes/TIAs (1.3%) was low and did not differ statistically between study arms.
- The effect of CLS on AHRE reduction was observed in patients without atrioventricular block (23% relative risk reduction; *P* = 0.008) and without history of atrial fibrillation (27% relative risk reduction; *P* = 0.009).

## Introduction

Subclinical atrial fibrillation (AF), diagnosed by cardiovascular implantable electronic devices as atrial high-rate episodes (AHREs) lasting for a minimum of 5–6 min in patients without history of AF, is associated with an increased risk of progression to clinical AF (symptomatic or documented by surface electrocardiogram), stroke, heart failure hospitalization, and cardiovascular death.^1–7^ In patients with an indication for dual-chamber pacing, the search for an optimal pacing strategy to prevent or delay the development and progression of subclinical AF may have clinical relevance.

For the treatment of sinus node dysfunction (SND) and chronotropic incompetence, the latest European Society of Cardiology guidelines recommend dual-chamber rate-adaptive (DDDR) pacing.^8^ The accelerometer is the most common sensor for rate-adaptive pacing due to its simplicity and reliability. Closed loop stimulation (CLS) is an alternative approach, integrated into the circulatory control system through intra-cardiac impedance monitoring and responding to increased metabolic demand, mental stress, and autonomic stressors independently of body movements or acceleration.^9–15^ Small short-term studies have indicated a potential benefit of CLS in the reduction of atrial tachyarrhythmia burden compared with alternative pacing strategies.^16–18^

To further investigate this issue, we conducted a formal randomized trial in a large cohort of patients with pacemakers and implantable cardioverter-defibrillators (ICDs) followed for 3 years after randomization to dual-chamber CLS or accelerometer-based DDDR pacing. The primary outcome measure was time to first AHRE ≥ 6 min, stroke, or transient ischaemic attack (TIA).

## Methods

### Study design and patient selection

The Clinical Benefits of the Closed Loop Stimulation in Sinus Node Disease (‘B3’ study) was a multi-centre, randomized, single-blind trial including patients with a Class I or II indication for permanent pacing due to SND with or without atrioventricular (AV) block, for whom dual-chamber rate-responsive pacing was indicated or preferred and antiarrhythmic drug therapy was optimized and stable at the time of enrolment. The study protocol definition of SND was according to guidelines,^8^ which included a wide spectrum of persistent or intermittent symptomatic sino-atrial dysfunctions, ranging from sinus bradycardia, sino-atrial block, and sinus arrest to bradycardia–tachycardia syndrome, with the additional manifestation of inadequate chronotropic response to exercise. No exercise tests were required by the study protocol at baseline. Enrolled patients had a recently implanted pacemaker or ICD (Biotronik SE & Co. KG, Berlin, Germany) offering dual-chamber CLS mode, accelerometer-based DDDR mode, and diagnostic memory for atrial and ventricular arrhythmia episodes including associated intra-cardiac electrograms (IEGMs).

Major exclusion criteria were permanent AF, recent AF ablation or cardiac surgery, an indication for cardiac resynchronization therapy, New York Heart Association Class IV heart failure, Stage V renal disease, life expectancy of <1 year, and age < 18 years. Patients were also excluded if device implantation was not *de novo*, a stroke occurred since implantation, CLS was activated before enrolment, or antiarrhythmic therapy was not optimized and stable.

All enrolled patients provided written informed consent. Relevant national and local ethics committees approved the study protocol (clinical sites are listed in the section ‘Investigators and other parties’ in Supplementary material online, *Appendix*). The study was conducted in accordance with the guidelines for good clinical practice and the Declaration of Helsinki (ClinicalTrials.gov Identifier: NCT02579889).

### Study protocol

Device implantation was not part of the study, and investigational sites performed it according to their institutional standards. Ventricular leads were implanted in the right ventricle; conduction system pacing (His bundle or left bundle branch area pacing) was not used. The enrolment visit occurred at any time between implantation and randomization, to assess patient eligibility and collect their demographic data, medical data, and signed informed consents.

At the randomization visit (90 ± 30 days after implantation), patients in sinus rhythm were randomly assigned (1:1) to dual-chamber CLS mode or accelerometer-based DDDR mode. Patients with an ongoing AF episode were excluded and replaced. The randomization was done through a centralized process implemented by the sponsor and stratified by site, device type (pacemaker/ICD), and AV block (yes/no). The random allocation sequence with variable and randomized block size (from 2 to 8) was computer generated and concealed from the sites. Randomization was communicated to the sites on a dedicated webpage with electronic case report forms. Investigators were not masked to treatment allocation. Patients were masked to treatment allocation until the end of the study.

Pacemakers and ICDs were programmed according to recommendations in study protocol, including AHRE detection rate of 190 b.p.m., basic pacing rate of 60 b.p.m., upper rate of 130 b.p.m., dynamic AV delay ON, and an AV hysteresis controlled by the IRS^plus^ algorithm (favouring intrinsic AV conduction).^19^Supplementary material online, *Table S1* lists all programming recommendations.

Patients were followed for 3 years after randomization, with hospital visits scheduled at 1, 2, and 3 years (see Supplementary material online, *Figure S1*). During scheduled and unscheduled hospital visits, diagnostic data were retrieved from the implanted devices, including the onset date/time, duration, and atrial and ventricular IEGM recording relative to the first episode since the last interrogation, the longest episode, and all most recent episodes until memory saturation (depending on device capacity). The investigators also examined patients physically and documented medical history, cardiovascular medication, and adverse events.

### Primary endpoint and study hypotheses

The primary endpoint of the study was time to the composite endpoint of the first AHRE lasting for at least 6 min, stroke, or TIA. Stroke was defined as a focal neurologic deficit of sudden onset, as would be expected with occlusion of one of the major cerebral arteries, which did not resolve within 24 h. The primary study hypothesis was that a 3-year freedom from the primary endpoint would be higher in the CLS arm than in the DDDR arm on the intention-to-treat basis. The corresponding null hypothesis is that there is no difference.

A breakdown of the primary endpoint to four strata of AHRE duration (6 min to <6 h, 6 h to <24 h, 24 h to <7 days, and ≥7 days) and stroke or TIA was analysed. Secondary endpoints were AHRE ≥ 6 min, AHRE ≥ 7 days (i.e. persistent AF), permanent AF (arrhythmia accepted by the patient and the physician), stroke or TIA, hospitalization for worsening heart failure with at least one overnight stay, and all-cause death.

A *post hoc* exploratory analysis compared the incidence of AHRE ≥ 6 min between the two study arms in predefined subgroups of patients dichotomized by sex (male/female), age (<76/≥76 years), resting heart rate (<70/≥70 b.p.m.), AV block (no/yes), percentage of ventricular pacing (<20%/≥20%), history of AF (no/yes), CHA_2_DS_2_-VASc score (<3/≥3), diabetes mellitus (no/yes), and congestive heart failure (no/yes).

### Endpoint adjudication board

All events potentially contributing to endpoints were verified by an independent three-member Endpoint Adjudication Board masked to treatment allocation (section 1.3. in Supplementary material online, *Appendix*). Adjudication of AHREs was done according to a pre-specified charter by visual inspection of atrial and ventricular IEGM recordings and associated diagnostic data. In case of divergent opinion of the first two adjudicators, the decision was made by majority vote.

### Statistical methods

The study was designed to detect an absolute reduction in the 3-year incidence of the primary endpoint in the CLS arm of 2.4%, with a Type I error of *α* = 0.05 (bilateral) and a statistical power of 80%. This was based on an expected 1-year incidence of the primary endpoint of 8% in the DDDR arm (obtained by combining literature data in an exponential distribution model). Assuming an early dropout of ≤10% and a 2% loss of power due to interim analyses described below, the number of enrolled patients had to be 1308 (654 per study arm). The required number of primary endpoint events was 230.

Three interim analyses and the final analysis were planned after 25%, 50%, 75%, and 100% of primary endpoint events, respectively. The Steering Committee (section 1.2. in Supplementary material online, *Appendix*) evaluated the results of the interim analyses to consider early termination of the study for efficacy, futility, or low conditional power based on whether the *Z*-test values exceeded the pre-specified limits (sequential O’Brien–Fleming boundaries, Supplementary material online, *Table S2*). The final adjusted inference after interim analyses was based on stagewise ordering of the sample space.^20,21^

We compared the treatment groups on the intention-to-treat basis. In the time-to-event analyses, we used Cox proportional hazard models stratified by site and the Kaplan–Meier method to determine the hazard ratio (HR) and 95% confidence interval (CI). For the primary endpoint, the HR was calculated unadjusted and adjusted by the CHA_2_DS_2_-VASc score. As a sensitivity analysis, competing-risk regression analysis for the primary endpoint was performed clustering observations by investigational sites for standard errors, using CLS treatment as independent variable and all-cause death as competing risk. Cumulative incidence functions were plotted by study groups after fit of the competing-risk regression model. All analyses were repeated using a per-protocol approach.

Continuous variables are reported as median with interquartile range (IQR). Percentages of atrial pacing and ventricular pacing in the two treatment groups were compared using the Mann–Whitney *U* test. Binary or categorical variables are reported as counts and percentages of non-missing data.

For the primary endpoint, nominal *P*-values at interim and final analyses were determined according to the pre-specified sequential design to control for an overall bilateral Type I error of *α* < 0.05. In all other analyses, a two-sided *P*-value of <0.05 was considered statistically significant. The analysis was carried out by the Clinical Research Unit of Biotronik Italia S.p.A. (Cologno Monzese, MI, Italy) and blindly validated at the Unit of Biostatistics, University of Padua, Italy (section 1.4. in Supplementary material online, *Appendix*). The statistical software packages used were the STATA 18.0/MP (StataCorp, TX, USA) and R version 4.2.3 (The R Foundation for Statistical Computing Platform, Vienna, Austria).

## Results

### Patient population

**Figure 1:**
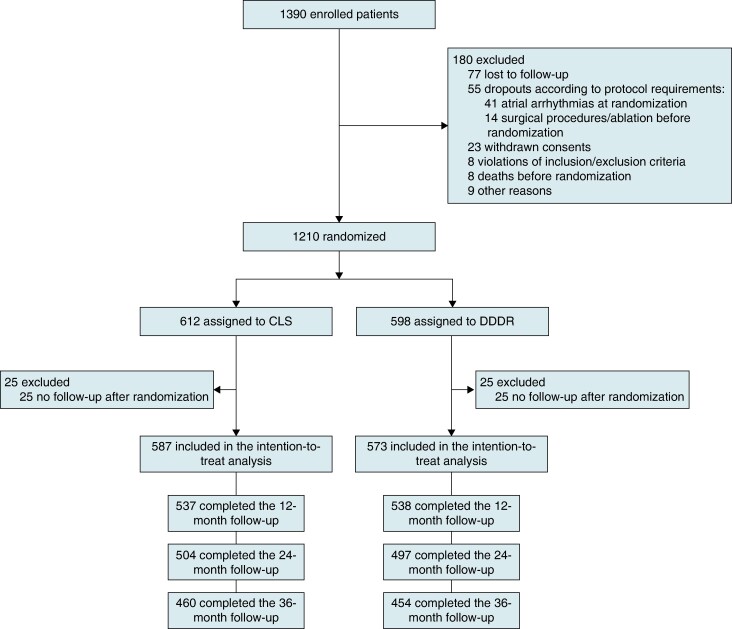
Trial flow chart (CONSORT diagram). All patients had an implanted device at enrolment. The randomization visit took place 90 ± 30 days after implantation. CLS, closed loop stimulation; DDDR, dual-chamber rate-adaptive.

From September 2015 to September 2019, a total of 1390 patients were enrolled at 53 clinical centres in Europe and Asia (see section 1.1. in Supplementary material online, *Appendix*). After the exclusion of 180 patients for the reasons explained in the CONSORT chart (*Figure 1*), 1210 patients were randomly assigned to CLS (*n* = 612) or DDDR mode (*n* = 598).

There were no notable differences in patient characteristics between the two study arms (*Table 1*). Median patient age was 76 years (IQR, 70–81), 47.2% were women, 97.7% received pacemaker therapy, and 2.3% received ICD therapy. More than one-third of patients (36.2%) had a history of atrial arrhythmia, mostly paroxysmal AF, and anticoagulation was prescribed in 36.1%. Median CHA_2_DS_2_-VASc score was 3 (IQR, 2–4). About one-third of patients had AV block (29.2%), and relatively few had congestive heart failure (18.4%).

**Table 1:** Baseline characteristics of randomized patients

Characteristic at enrolment	CLS arm (*n* = 612)	DDDR arm (*n* = 598)	All patients (*n* = 1210)
Age (years)	76 [71–82]	76 [69–81]	76 [70–81]
Female sex	298 (48.8)	273 (45.7)	571 (47.2)
Body mass index (kg/m^2^)	26 [23–29]	26 [24–29]	26 [23–29]
Implanted device			
Dual-chamber pacemaker	597 (97.5)	585 (97.8)	1182 (97.7)
Dual-chamber ICD	15 (2.5)	13 (2.2)	28 (2.3)
Sinus node dysfunction	612 (100)	598 (100)	1210 (100)
Main indication for pacing:			
Bradycardia-tachycardia form	222 (37.3)	220 (37.6)	442 (37.4)
Sinus bradycardia/chronotropic incompetence	209 (35.1)	210 (35.9)	419 (35.5)
Sinus arrest	106 (17.8)	91 (15.6)	197 (16.7)
Sino-atrial block	59 (9.9)	64 (10.9)	123 (10.4)
Atrioventricular block			
None	431 (70.4)	426 (71.2)	857 (70.8)
First degree	104 (17.0)	88 (14.7)	192 (15.9)
Second degree, Type I	37 (6.0)	45 (7.5)	82 (6.8)
Second degree, Type II	25 (4.1)	20 (3.3)	45 (3.7)
Third degree	15 (2.5)	19 (3.2)	34 (2.8)
History of AF	213 (34.9)	223 (37.6)	436 (36.2)
Medical history			
Hypertension	428 (70.0)	417 (70.0)	845 (70.0)
Diabetes mellitus	128 (20.9)	135 (22.7)	263 (21.8)
Congestive heart failure	104 (17.0)	118 (19.8)	222 (18.4)
Abnormal renal function^a^	41 (6.7)	46 (7.7)	87 (7.2)
Stroke or transient ischaemic attack	45 (7.4)	38 (6.4)	83 (6.9)
Left ventricular ejection fraction (%)	59 [55–62]	60 [55–62]	60 [55–62]
Risk scores			
Virtual^b^ CHA_2_DS_2_-VASc (scale: 0 to 9 [highest risk])	3 [2–4]	3 [2–4]	3 [2–4]
ATRIA (scale: 0–6 [highest risk])	2 [1–3]	2 [1–3]	2 [1–3]
HAS-BLED (scale: 0–9 [highest risk])	2 [1–3]	2 [1–3]	2 [1–3]
Medications			
Oral anticoagulant	222 (36.3)	214 (35.9)	436 (36.1)
Beta-blocker	211 (34.5)	214 (35.9)	425 (35.2)
Antiplatelet	213 (34.9)	195 (32.7)	408 (33.8)
Diuretic	200 (32.7)	193 (32.4)	393 (32.6)
Calcium antagonist	164 (26.8)	166 (27.9)	330 (27.3)
ACE inhibitor	165 (27.0)	146 (24.5)	311 (25.8)
Antiarrhythmic	62 (10.1)	66 (11.1)	128 (10.6)
Ia type	7 (11.3)	10 (15.4)	17 (13.4)
Ib type	1 (1.6)	1 (1.5)	2 (1.6)
Ic type	54 (87.1)	54 (83.1)	108 (85.0)
Amiodarone	61 (10.0)	50 (8.4)	111 (9.2)
Sotalol	12 (2.0)	7 (1.2)	19 (1.6)
Device diagnostics at randomization			
Percentage of atrial pacing (%)	56 [24–83]	56 [24–26]	56 [25–81]
Percentage of ventricular pacing (%)	3 [0–35]	3 [0–27]	3 [0–29]
Atrial arrhythmia burden (% of time)	0 [0–0]	0 [0–0]	0 [0–0]
Patients with atrial arrhythmia burden > 0	95 (15.7)	111 (18.7)	206 (17.2)

Data are median [IQR] or *n* (% of available data).

ACE, angiotensin-converting enzyme; AF, atrial fibrillation; ATRIA, bleeding risk in AF; CHA_2_DS_2_-VASc, the score including **C**ongestive heart failure (1 point), **H**ypertension (1 point), **A**ge ≥75 years (2 points), **D**iabetes (1 point), previous **S**troke or TIA (2 points), **V**ascular disease (1 point, e.g. myocardial infarction), **A**ge 65–74 years (1 point), and female **S**ex **c**ategory (1 point); CLS, closed loop stimulation; DDDR, dual-chamber rate-adaptive; HAS-BLED, 1-year risk of major bleeding in patients taking anticoagulants for AF; ICD, implantable cardioverter-defibrillator.

^a^Chronic dialysis, renal transplantation, glomerular filtration rate < 30 mL/min, or serum creatinine > 200 umol/L.

^b^‘Virtual’ indicates that the score was calculated irrespective of whether the patient has or does not have AF.

### Follow-up

After randomization, 50 patients were lost to follow-up and the remaining 1160 patients contributed to the intention-to-treat analysis. Incorrect programming of the randomly allocated pacing mode occurred in 72 patients (55 in the CLS arm and 17 in the DDDR arm), and they were excluded from the per-protocol analysis.

The study was terminated early on 10 February 2023, in response to the Endpoint Adjudication Board’s announcement that the required number of primary endpoint events to test the primary study hypothesis had been reached. Previously, two interim analyses were carried out, and the third was skipped to initiate the study termination procedure. All endpoint-related events that occurred through February 10 were adjudicated, and the final analysis was conducted. The nominal *P*-value of <0.049, calculated based on two interim analyses, was required for statistical significance in the final analysis (see Supplementary material online, *Tables S3* and *S4*).

The numbers of patients completing the 1-, 2-, and 3-year follow-ups before study termination were 1075, 1001, and 914, respectively (*Figure 1*). The cumulative follow-up time was 3186 patient-years.

### Total number of atrial high-rate episodes and thromboembolic events

Of 7332 AHREs detected by implanted devices and documented by IEGM recordings during the study, 7236 (98.7%) were classified as true positive by the Endpoint Adjudication Board, of which 6871 (93.7%) had irregular form, 241 (3.3%) were atrial flutter, and 124 (1.7%) were atrial tachycardia. False-positive AHRE detections (*n* = 96) were mainly caused by electrical noise from potential lead failure (*n* = 40), electromagnetic interference (*n* = 32), and far-field R-wave over-sensing (*n* = 14) (see Supplementary material online, *Tables S5* and *S6*).

The total number of cerebrovascular thromboembolic events was 17, including 12 strokes (11 ischaemic and 1 haemorrhagic) and five TIAs, affecting 15 patients. Eight ischaemic strokes/TIAs occurred before AHREs ≥ 6 min and were therefore included in the primary endpoint analysis. The remaining nine strokes/TIAs were preceded by AHRE(s) ≥ 6 min and were therefore no primary endpoints.

### Primary endpoint

The primary endpoint occurred in 50.6% of patients in the CLS arm (297/587) and 55.7% of patients in the DDDR arm (319/573). The final unbiased estimate of HR was 0.84 (95% CI, 0.72–0.99), using the site-stratified Cox regression model (number needed to treat to prevent the endpoint in one patient, 22.2). The *P*-value of 0.035 is lower than the required level of 0.049 to reject the primary null hypothesis. The difference between the two study arms remained significant after adjusting for the CHA_2_DS_2_-VASc score (HR, 0.84; 95% CI, 0.71–0.99; *P* = 0.033) and including all-cause deaths as competing risk (sub-HR, 0.85; 95% CI, 0.74–0.98; *P* = 0.028). The results were confirmed also by the sensitivity analyses on the intention-to-treat and per-protocol basis (see Supplementary material online, *Tables S7* and *S8* and *Figure S2*).

Breakdown of the primary endpoint in *Table 2* shows that the difference between the two study arms was mainly driven by AHREs, particularly episodes lasting between 6 h and 7 days, which accounted for 30.7% of primary endpoint events (CLS) vs. 35.4% (DDDR). Short AHREs (≥6 min to <6 h) were the most frequent primary endpoint event in both study arms, 61.3% (CLS) vs. 59.9% (DDDR), affecting 31.0% (CLS) and 33.3% (DDDR) of patients. The overall median duration of AHREs tended to be shorter in CLS (2.6 vs. 3.0 h). No patient had permanent AF as the first arrhythmic episode, while the contribution of stroke or TIA to the primary endpoint was small in both study arms: 1.7% (CLS) vs. 0.9% (DDDR).

**Table 2:** Breakdown of the primary endpoint in the analysis population

Event (intention-to-treat analysis)	CLS arm (587 patients)	DDDR arm (573 patients)	% of patients	
			CLS arm	DDDR arm
Primary endpoint events	297	319	50.6	55.7
Atrial high-rate episodes				
≥ 6 min to <6 h	182 (61.3)	191 (59.9)	31.0	33.3
6 h to <24 h	65 (21.9)	82 (25.7)	11.1	14.3
24 h to <7 days	26 (8.8)	31 (9.7)	4.4	5.4
≥7 days	19 (6.4)	12 (3.8)	3.2	2.1
Permanent	0 (0)	0 (0)	0.0	0.0
Stroke or transient ischaemic attack	5 (1.7)	3 (0.9)	0.9	0.5
Duration of atrial high-rate episode (h)	2.6 [0.6–10.9]	3.0 [0.6–11.1]	—	—

Data are number of events (% of the total number of primary endpoint events), % of patients, or median [IQR].

CLS, closed loop stimulation; DDDR, dual-chamber rate-adaptive.

### Secondary endpoints, atrial high-rate episode burden, and atrial pacing percentage

**Figure 2:**
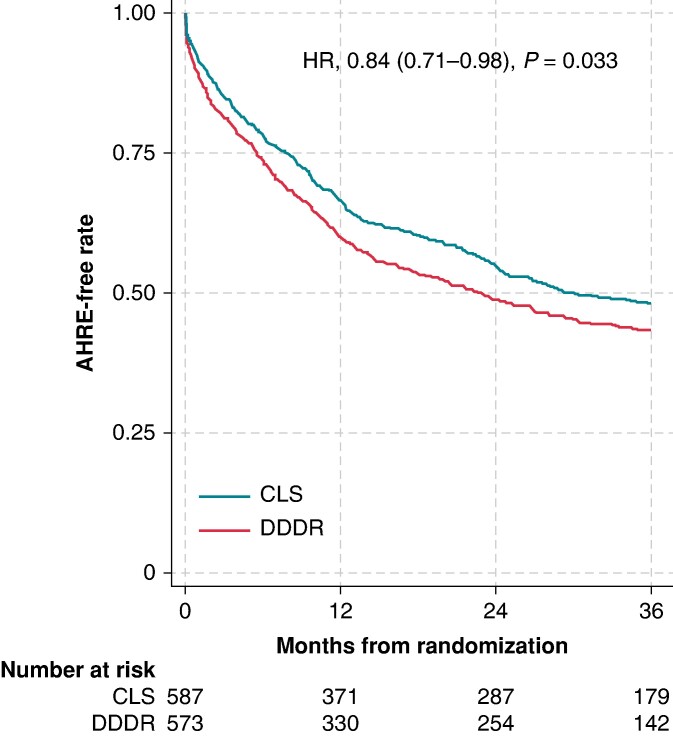
Kaplan–Meier estimate for the incidence of AHRE ≥ 6 min by study arm on the intention-to-treat basis. *P*-value was calculated using Cox proportional hazard models stratified by site. AHRE, atrial high-rate episode; CI, confidence interval; CLS, closed loop stimulation; DDDR, dual-chamber rate-adaptive; HR, hazard ratio.

Secondary endpoints are compared in *Table 3*. Statistical significance was reached for AHRE ≥6 min (HR in CLS, 0.84; 95% CI, 0.71–0.98; *P* = 0.033; *Figure 2*), which was confirmed by per-protocol analysis (see Supplementary material online, *Table S9*). Statistical power was insufficient to detect differences in other secondary endpoints.

**Table 3:** Secondary endpoints

Events (intention-to-treat analysis)	CLS arm (587 patients)	DDDR arm (573 patients)	Hazard ratio (95% CI)	*P*-value	Statistical power
AHRE, *n* (%)					
≥ 6 min	293 (49.9)	316 (55.2)	0.84 (0.71–0.98)	0.033	67%
≥ 7 days	55 (9.4)	46 (8.0)	1.21 (0.81–1.81)	0.35	14%
Permanent AF	29 (4.9)	19 (3.3)	1.53 (0.83–2.80)	0.17	37%
Stroke or TIA	10 (1.7)	5 (0.9)	1.96 (0.67–5.77)	0.22	26%
Hospitalization for WHF	16 (2.7)	10 (1.8)	1.70 (0.77–3.78)	0.19	22%
All-cause death	38 (6.5)	41 (7.2)	0.84 (0.53–1.33)	0.46	6%

Data are *n* (% of patients) unless otherwise stated.

AF, atrial fibrillation; AHRE, atrial high-rate episode; CI, confidence interval; CLS, closed loop stimulation; DDDR, dual-chamber rate-adaptive; TIA, transient ischaemic attack; WHF, worsening heart failure.

Calculation of AHRE burden was not foreseen in the study design based on a time-to-first episode analysis with 3-year follow-up and because this metric cannot be fully assessed by IEGM documentation. Atrial high-rate episode burden is provided by the devices as the cumulative time percentage (1% burden in 1 year ≈ 3.7 days) with detected high atrial rate above a programmable cut-off value (190 b.p.m. in our study). The median 1-year AHRE burden was 0% in both study arms (average 4.7% vs. 5.3%, 90th centile, 5% vs. 6% in the CLS vs. DDDR arm).

**Figure 3:**
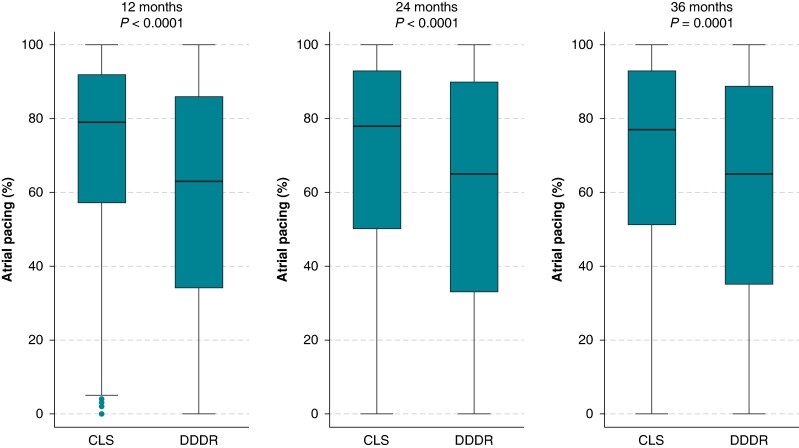
Percentage of atrial pacing in the two study arms. Data are shown as box plot with boxes representing median values (inner lines) and the IQR and whiskers representing adjacent value ranges (1.5 times the range of the nearest quartile). CLS, closed loop stimulation; DDDR, dual-chamber rate-adaptive.

The median percentage of atrial pacing was consistently higher in the CLS arm (77–79%) than in the DDDR arm (63–65%) during the 3-year follow-up period (*P* < 0.001; *Figure 3*).

### Subgroup analyses of atrial high-rate episode incidence

**Figure 4:**
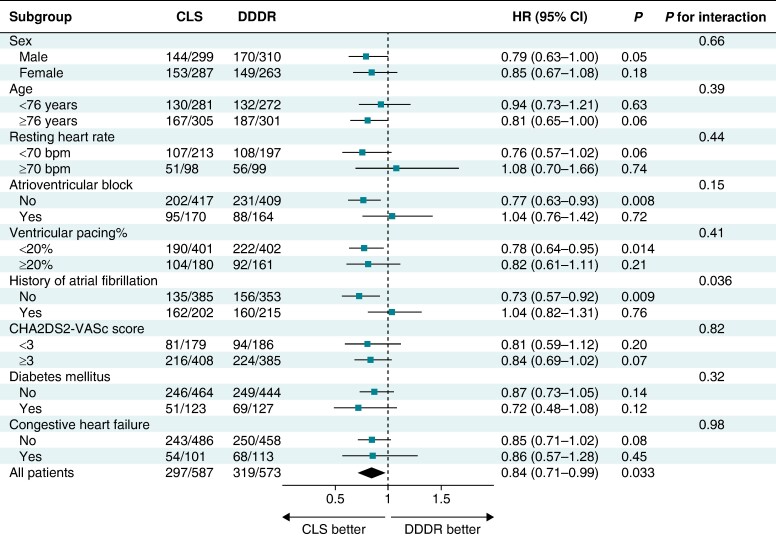
Subgroup analyses of AHRE ≥ 6 min stratified by site (intention-to-treat). For CHA_2_DS_2_-VASc, see *Table 1*. CI, confidence interval; HR, hazard ratio.

**Figure 5:**
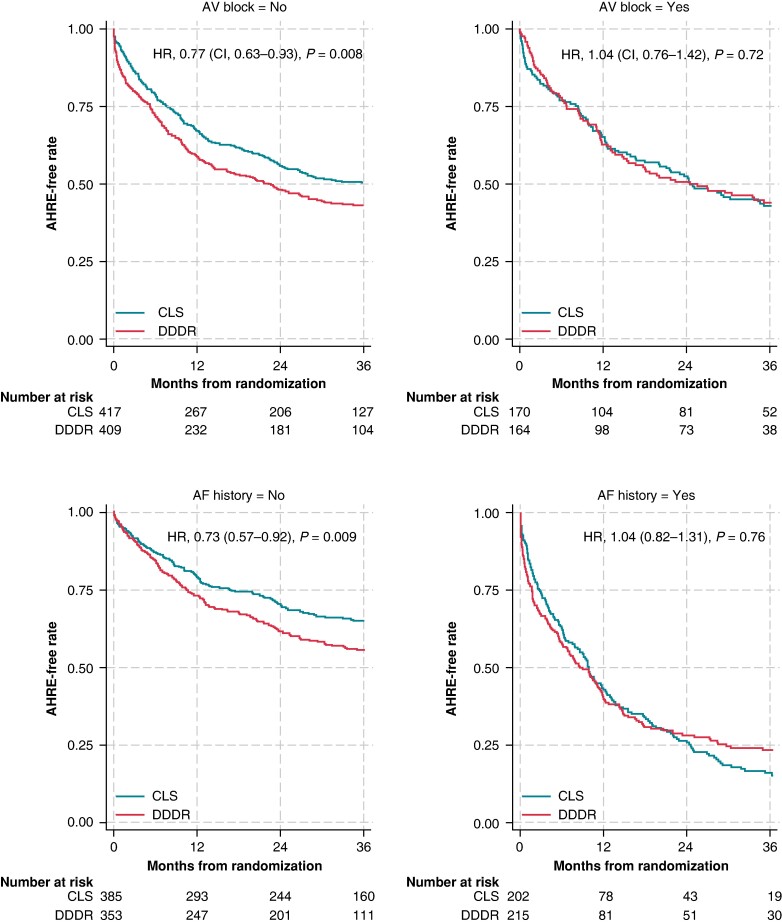
Kaplan–Meier estimate for the incidence of AHRE ≥ 6 min in patients without vs. with AV block (*A*), and in patients without vs. with history of AF (*B*). *P*-values were calculated using Cox proportional hazard models stratified by site. AF, atrial fibrillation; AHRE, atrial high-rate episode; AV, atrioventricular; CI, confidence interval; CLS, closed loop stimulation; DDDR, dual-chamber rate-adaptive; HR, hazard ratio.

In a *post hoc* analysis of AHRE incidence by subgroups (*Figure 4*), the incremental effect of CLS was greatest in patients without AV block (HR, 0.77; *P* = 0.008) or without history of AF (HR, 0.73; *P* = 0.009). *Figure 5* illustrates significant differences in these subgroups and neutral findings in the complementary subgroups. *Figure 5B* also shows that 54–63% of patients without history of AF and only 14–21% of patients with AF history were free from AHREs at 3 years. Consistent with the results for no AV block, the incremental benefit of CLS was greater for <20% of ventricular pacing (HR, 0.78; *P* = 0.014) (*Figure 4*).

### Adverse events

Thirteen patients (1.1%) experienced serious device-related complications, including lead dislodgment (*n* = 9), lead failure (*n* = 2), and pocket infection/decubitus necessitating surgical revision (*n* = 2). Palpitations in the CLS mode were reported by six patients (1.0%) and resolved by reprogramming the CLS response factor or maximum pacing rate (*n* = 3) or by switching to dual-chamber pacing without rate-adaptive function (*n* = 3).

## Discussion

Our study of SND patients followed for 3 years after randomization showed that dual-chamber CLS mode was associated with a significantly lower incidence of AHRE ≥ 6 min, as compared with the DDDR mode. This difference was mostly driven by fewer episodes lasting between 6 h and 7 days in the CLS arm. Patients without AV block (i.e. with low ventricular pacing) and patients without history of AF had the greatest incremental benefit of CLS. The incidence of stroke/TIA, heart failure hospitalization, or all-cause death was low and did not differ between the two pacing strategies.

### The relevance of atrial high-rate episodes according to current evidence

The clinical relevance of AHREs is one of the main topics of current scientific debate.^7,22–25^ Atrial high-rate episodes are frequently detected even in patients without known AF: the reported 2-year incidence with implantable devices is 30–35% for episodes ≥ 6 min and 4–10% for episodes ≥ 24 h.^26–28^ We observed AHREs ≥ 6 min in 49.9% of patients in the CLS arm and 55.2% in the DDDR arm after 3-year follow-up. The increasing adoption of wearable devices will inevitably contribute to AHRE detection in a growing population, with potential consequences for healthcare systems if appropriate screening and treatment strategies are not clarified. In relation to thromboembolic risk, AHREs are associated with up to 2.5-fold higher risk of stroke, depending on AHRE burden and the CHA_2_DS_2_-VASc score,^1,29–33^ which is, however, lower than the overall five-fold increased risk expected in clinical AF.^33^ Also, thromboembolic events appear temporally disconnected from AHRE recurrences, showing that AHREs should be considered as a marker of cardioembolic risk rather than a mechanical cause of stroke.^30,34^ The recent results of The Atrial Fibrillation Detected by Continuous Electrocardiogram Monitoring Using Implantable Loop Recorder to Prevent Stroke in High-Risk Individuals (LOOP) study^35^ have even questioned the importance of long-term continuous AF screening with implantable loop recorders in patients with stroke risk factors and no indication for cardiac pacing or ICD. Although AHRE detection and initiation of oral anticoagulation were three times more common in the implantable loop recorder group compared with controls, no difference in stroke incidence was observed between groups.^35^

Very recently, The Apixaban for the Reduction of Thrombo-Embolism in Patients with Device-Detected Subclinical Atrial Fibrillation (ARTESiA)^3^ trial and The Non–Vitamin K Antagonist Oral Anticoagulants in Patients with Atrial High Rate Episodes (NOAH-AFNET 6)^2^ trial have consistently shown that in a population predominantly using pacemakers as well as ICDs and implantable loop recorders, and with previously documented AHREs ≥ 6 min (average duration, 1.5–3.0 h), the use of novel oral anticoagulants reduces the risk of stroke but increases the risk of major bleeding,^36^ which may be of significant concern. Interestingly, the annual rate of ischaemic stroke reported in the control arms of both the ARTESiA and NOAH-AFNET 6 trials was lower than expected (≈1.0%). This is consistent with the annual stroke rate of 0.5% observed in our study population with a lower risk profile (mean CHA_2_DS_2_-VASc score 3 vs. 4) and no history of atrial tachyarrhythmias in the majority (two-thirds) of cases. The very low incidence of ischaemic strokes on the one hand and the increase in haemorrhagic events due to anticoagulation on the other hand leave many questions unanswered about the role and net clinical benefit of novel oral anticoagulants in patients with AHREs. A risk management based on shared decision-making with patients appropriately informed about the associated risks and benefits has recently been proposed as a reasonable approach in such a context.^37^ Nevertheless, the uncertainties in the prevention of thromboembolic risk do not undermine the importance of AHRE monitoring in routine medical practice: short AHREs remain the strongest predictor of long-lasting episodes and clinical AF^32,38^ (which *per se* implies a five-fold higher risk of stroke^33,38^) and contribute to a 4.6-fold increased risk of heart failure hospitalization compared with the absence of AHREs.^6,7^ Cardiovascular and stroke mortality has also been reported to be associated with AHREs in some studies.^5,39^ Therefore, AHREs likely tag worsening patient conditions,^40^ and any strategy to prevent new onset or progression of AHREs is warranted.^22^

### Findings of the present study

Our large, randomized trial with fully adjudicated AHRE data demonstrated the effect of CLS in a time-to-event analysis in patients with SND who predominantly had no known AF (63.8%). The observed reduction in AHRE incidence was small but primarily caused by fewer episodes lasting between 6 h and 7 days in the CLS arm. These episodes are considered more clinically relevant than shorter episodes, although a clear cut-off point for duration has not been definitely identified.^22,33,41^ Nevertheless, in our population characterized by a moderate risk profile, we failed to detect any difference between CLS and DDDR pacing in terms of persistent (≥7 days) or permanent AF, heart failure hospitalizations, strokes/TIAs, and deaths. We observed a limited rate of major cardiovascular events during the 3-year follow-up period, preventing meaningful evaluation of these endpoints. In particular, the incidence of stroke/TIA was much lower than expected. Also, we observed no benefit in the subset of patients with AF history and in patients with AV block requiring frequent right ventricular pacing. This is consistent with the well-established notions of AHRE progression and cardiomyopathy induced by pacing or dyssynchrony.^42^ On one hand, these results suggest that maximum benefit associated with CLS may be obtained in SND patients with no history of AF. On the other hand, the emerging evidence of preserved synchrony of ventricular contraction by conduction system pacing^43^ may encourage further research on the combined effect of CLS and conduction system pacing even in patients with AV block and/or frequent ventricular pacing.^44^

### Potential mechanism of closed loop stimulation benefit

Our results are in line with a few smaller randomized or retrospective studies conducted in the past with a shorter follow-up and non-adjudicated or partly adjudicated AHREs, which reported a reduction in AHRE burden with CLS as compared with DDDR or an atrial overdrive algorithm (see discussion in Supplementary material online, *Appendix*).^16–18^ However, the exact mechanism by which CLS suppresses new-onset or recurrent AHREs better than conventional DDDR pacing is not clear. Closed loop stimulation functioning is based on the continuous monitoring of impedance trends during each systolic phase (see Supplementary material online, *Figure S3*). Closed loop stimulation responds to variations in impedance trends by adjusting the pacing rate in each cardiac cycle. Since the impedance trend within each systole is correlated with cardiac contractility, CLS has been shown to detect and react not only to physical activity^45^ but also to active standing, handgrip, cold pressor test, mental stress, and dobutamine infusion.^9–15,46^ Under equal conditions, higher percentages of atrial pacing are therefore expected from CLS than from an accelerometer-based rate-responsive system. However, it does not seem plausible that the reduced incidence of AHRE with CLS observed in our study is only due to increased proportion of atrial pacing (that was consistently higher in the CLS arm than in the DDDR arm during the 3-year follow-up period). Other algorithms specifically designed to maximize atrial pacing have been tested in the past, but failed to reduce AHRE occurrence and burden.^1,47,48^ More recently, even the reverse hypothesis that minimizing atrial pacing can reduce the incidence of AHREs was tested in The Danish Multicenter Randomized Study on DDD-40 vs. DDDR-60 Pacing in Sick Sinus Syndrome (DANPACE II), but dual-chamber mode with 40 b.p.m. base rate was not superior to DDDR mode with 60 b.p.m. base rate.^49^ The small but statistically significant reduction in AHRE incidence observed with CLS in comparison with conventional DDDR pacing may therefore be related to the operating principle of CLS (see CLS description in Supplementary material online, *Appendix*) and to a combined effect of increased atrial pacing percentage and higher heart rates in response to a wider range of metabolic demands compared with the accelerometer-based rate-adaptive pacing. However, specifically designed investigations are needed to assess such hypothesis.

### Study limitations

The primary composite endpoint of time to first AHRE ≥ 6 min, stroke, or TIA implies the use of AHRE as a surrogate for ischaemic thromboembolism. This seemed reasonable at the time of study design but proved suboptimal in light of the recent evidence discussed above.^2,3,28,32^ Second, although dual-chamber rate-responsive pacing was indicated or preferred according to guidelines in all enrolled patients, the study protocol did not require any specific exercise test to assess chronotropic incompetence at baseline. Furthermore, only AHREs validated by adjudication of IEGM recordings contributed to study results. Due to the relatively high incidence of AHRE detections in our population and inevitable limitations in device memory capacity, it was not possible to ensure collection of IEGMs for all AHRE recurrences after the first (or longest) episodes since the last device interrogation. Therefore, we planned only the time-to-first-event analysis and not recurrent-event analysis that would be able to provide more information on arrhythmia progression and AHRE burden. Even the Home Monitoring technology integrated in the implanted devices (and used in a minority of study patients) cannot ensure transmission of IEGMs of all detected AHREs. In addition, significantly more IEGM-documented AHREs occurred during the study than expected. Based on the available literature at the time of study design, an annual incidence of AHREs of 8% was assumed, but the observed incidence was 31%, likely due to multiple evolving factors, such as device technology, diagnostic capacity, and scientific knowledge. Furthermore, due to the COVID-19 pandemic, a considerable number of scheduled hospital visits had to be postponed or replaced by phone contacts, which delayed the collection of AHREs and caused their over-accumulation prior to adjudication. This resulted in a delayed reporting of adjudicated endpoint events by the Endpoint Adjudication Board and in study prolongation.

## Conclusion

In our randomized clinical trial, about 50% of patients with a dual-chamber pacing system for SNDs experienced AHRE(s) ≥ 6 min during 3 years of follow-up. Compared with conventional rate-adaptive pacing, CLS mode was associated with a significantly lower incidence of the composite endpoint of first AHRE ≥ 6 min, stroke, or TIA, which was driven by lower AHRE incidence, especially in patients without AV block and without history of AF. The incidence of stroke/TIA, heart failure hospitalization, or all-cause death was low and did not differ between the two pacing strategies.

## Supplementary Material

euae175_Supplementary_Data

## Data Availability

The data underlying this article were provided by Biotronik SE & Co KG. Data will be shared upon reasonable request to the corresponding author with the permission of Biotronik SE & Co. KG.
