# Correction to: Closed loop stimulation reduces the incidence of atrial high-rate episodes compared with conventional rate-adaptive pacing in patients with sinus node dysfunctions

**DOI:** 10.1093/europace/euae217

**Published:** 2024-08-26

This is a correction to: Ennio C L Pisanò, Valeria Calvi, Miguel Viscusi, Antonio Rapacciuolo, Ludovico Lazzari, Luca Bontempi, Gemma Pelargonio, Giuseppe Arena, Vincenzo Caccavo, Chun-Chieh Wang, Béla Merkely, Lian-Yu Lin, Il-young Oh, Emanuele Bertaglia, Davide Saporito, Maurizio Menichelli, Antonino Nicosia, Domenico M Carretta, Aldo Coppolino, Chi Keong Ching, Álvaro Marco del Castillo, Xi Su, Martina Del Maestro, Daniele Giacopelli, Alessio Gargaro, Giovanni L Botto, Closed loop stimulation reduces the incidence of atrial high-rate episodes compared with conventional rate-adaptive pacing in patients with sinus node dysfunctions, *EP Europace*, Volume 26, Issue 7, July 2024, https://doi.org/10.1093/europace/euae175

In the originally published version of the manuscript, the graphical abstract image had typographical errors in the abbreviation “DDDR”. The errors have been emended throughout the Graphical Abstract.

The supplementary file was published with revisions visible. This has been replaced with all revisions accepted.

The emendations have been made to the article.

