# Supplementary material for: Closed loop stimulation reduces the incidence of atrial high-rate episodes compared with conventional rate-adaptive pacing in patients with sinus node dysfunctions
Source: Europace. 2024 Jun 28;26(7):euae175. doi: 10.1093/europace/euae175 (PMC11226787; doi:10.1093/europace/euae175)
Supplement: euae175_Supplementary_Data [file euae175_supplementary_data.docx]

**Supplementary Appendix**

This appendix has been provided by the authors to give readers additional information about their work.

Supplement to: Closed Loop Stimulation reduces the incidence of atrial high-rate episodes compared to conventional rate-adaptive pacing in patients with sinus node disease.

**Table of contents**

Page

1 Investigators and other parties …….…….…………………………………………………..... 3

1.1. Investigational sites ………………..….…….…………………………………………..... 3

1.2. Steering Committee ………………….….…………………………………………………. 4

1.3. Endpoint Adjudication Board ……………….…………………………………………. 4

1.4. Statistical analysis responsibility …………………………………………………...... 5

1.5. Funding ………………………………………..…………………………………………………. 5

1.6. Trial vigilance …………….……………………………………………………………………. 5

2 Supplementary methods ..…..………………..………………………………………………….. 6

2.1. Clinical investigation plan and amendment history …………………………. 6

2.2. Study flowchart ………………………………………………………………………………. 6

2.3. Device programming recommendations at randomization visit …...... 7

2.4. Group sequential design ……………………………………………………………....... 8

3 Supplementary results ………………...……..……..……..……..……………..….……..……. 9

3.1. Premature study termination ……………………………………………………….... 9

3.2. Endpoint adjudication …………………………………………………………………..... 10

3.3. Sensitivity analyses …….………………………………………………………………..... 11

4 Supplementary discussion …………...……..……..……..……..……………..….……..……. 14

4.1. Closed Loop Stimulation functioning principle ……………………………...... 14

4.2. Review of previous studies on CLS in sinus node disease………………..... 15

5 References ………………….……………...……..……..……..……..……………..….……..……. 16

Supplementary tables and figures Page

Table S1 …...........................................………………………………………………………. 7

Table S2 .............................................……………………………………………………….. 8

Table S3 .............................................……………………………………………………….. 9

Table S4 .............................................……………………………………………………….. 9

Table S5 .............................................……………………………………………………….. 10

Table S6 .............................................……………………………………………………….. 10

Table S7 .............................................……………………………………………………….. 11

Table S8 .............................................……………………………………………………….. 12

Table S9 .............................................……………………………………………………….. 12

Figure S1 .............................................……………………………………………………... 6

Figure S2 .............................................……………………………………………………… 13

Figure S3 .............................................……………………………………………………… 14

**1 Investigators and other parties**

**1.1. Investigational sites**

Investigational sites and number of enrolled patients (countries in alphabetical order, investigators in order of recruitment):

| Institution | Country | Principal investigator | N= |
| --- | --- | --- | --- |
| The 2nd Affiliated Hospital of Harbin Medical University | China | Bo Yu | 21 |
| Wuhan Asia Heart Hospital | China | Xi Su | 19 |
| Xuanwu Hospital | China | Yucong Zhang | 8 |
| Semmelweis University, Budapest | Hungary | Bela Merkely | 34 |
| Max Superspeciality Hospital | India | Viveka Kumar | 10 |
| Vito Fazzi Hospital, Lecce | Italy | Ennio C.L. Pisanò | 115 |
| Sant'Anna e San Sebastiano Hospital, Caserta | Italy | Miguel Viscusi | 97 |
| G. Rodolico - San Marco University Hospital, Catania | Italy | Valeria Calvi | 73 |
| AOU Federico II, Naples | Italy | Antonio Rapacciuolo | 68 |
| Santa Maria Hospital, Terni | Italy | Stefano Donzelli | 65 |
| Miulli Regional Hospital, Acquaviva delle Fonti | Italy | Vincenzo Caccavo | 58 |
| Fondazione Policlinico Gemelli IRCCS Università Cattolica del Sacro Cuore, Roma | Italy | Gemma Pelargonio | 50 |
| Infermi hospital, Rimini | Italy | Davide Saporito | 40 |
| Policlinico Consorziale, Bari | Italy | Domenico Carretta | 37 |
| Apuane New Hospital, Massa | Italy | Giuseppe Arena | 37 |
| AO Sant'Anna, Como | Italy | Carlo Piemontese | 34 |
| F. Spaziani Hospital, Frosinone | Italy | Maurizio Menichelli | 34 |
| F. Ferrari, Casarano | Italy | Donato Melissano | 33 |
| Bolognini Hospital, Seriate | Italy | Luca Bontempi | 32 |
| Maria S.S. Addolorata, Eboli | Italy | Angelo Catalano | 28 |
| Giovanni Paolo II Hospital, Ragusa | Italy | Antonino Nicosia | 27 |
| Ospedali Riuniti, Ancona | Italy | Antonio Dello Russo | 23 |
| AO dei Colli- Monaldi, Naples | Italy | Gerardo Nigro | 22 |
| SS. Annunziata Hospital, Savigliano | Italy | Aldo Coppolino | 22 |
| Camposampiero Hospital, Padova | Italy | Emanuele Bertaglia | 21 |
| AOU Maggiore della Carità, Novara | Italy | Gabriele Dell’Era | 18 |
| F. Veneziale, Isernia | Italy | Bruno Castaldi | 15 |
| S. Maria della Misericordia, Perugia | Italy | Gianluca Zingarini | 15 |
| AO Guido Salvini, Garbagnate | Italy | Fabio Locati | 15 |
| Cardarelli, Campobasso | Italy | Pierluigi Paolone | 14 |
| S. Maria della Stella, Orvieto | Italy | Andrea Mazza | 14 |
| Ospedale di Rho | Italy | Gianluca Botto | 14 |
| Sant'Antonio Abate, Gallarate | Italy | Salvatore Ivan Caico | 13 |
| ULSS7 - Ospedale di Conegliano | Italy | Giuseppe Allocca | 10 |
| Santa Maria Nuova, Firenze | Italy | Andrea Giomi | 8 |
| ULSS9 - Ospedale Treviso | Italy | Vittorio Calzolari | 7 |
| Nuovo Ospedale di Prato | Italy | Tiziana Giovannini | 4 |
| Policlinico Casilino, Roma | Italy | Ermenegildo De Ruvo | 2 |
| Seoul National Univ. Bundang Hospital (SNUBH) | Korea | Il-young Oh | 40 |
| Seoul National Univ. Hospital (SNUH) | Korea | Eue-Keun Choi | 23 |
| Bucheon Sejong Hospital | Korea | Sang-Weon Park | 14 |
| Korea University Anam Hospital | Korea | Jemin Shim | 9 |
| Pusan National University Yangsan Hospital | Korea | Ki Won Hwang | 7 |
| Soon Chun Hyang University Hospital | Korea | In Ki Moon | 1 |
| Hospital Sultan Idris Shah Serdang | Malaysia | Ahmad Fazli Abdul Aziz | 4 |
| National Heart Center Singapore (NHCS) | Singapore | Chi Keong Ching | 14 |
| Tan Tock Seng Hospital | Singapore | David Foo | 2 |
| Hospital Universitario 12 de Octubre, Madrid | Spain | Álvaro Marco del Castillo | 12 |
| Hospital General Universitario de Alicante | Spain | Juan Gabriel Martinez | 7 |
| Chang Gung Memorial Hospital -Linkou - CGMH | Taiwan | Chun-Chieh Wang | 39 |
| National Taiwan University Hospital | Taiwan | Lian-Yu Lin | 38 |
| National Cheng Kung University Hospital - NCKUH | Taiwan | Ju-Yi Chen | 22 |
| China Medical University Hospital - CMUH | Taiwan | Kuo-Hung Lin | 1 |
| **Total: 53 sites** | **9 countries** |  | **1390** |

**1.2. Steering Committee**

The Steering Committee has been responsible for the development of the clinical investigation plan, study amendments, monitoring of study progress and conduct, supervision of timing, evaluation of the results of interim and final analyses, and publication of results.

Members of the committee (alphabetical order):

- Giuseppe Arena Nuovo Ospedale Apuane, Marina di Massa MS, Italy
- Emanuele Bertaglia Ospedale di Camposampiero, Camposampiero PD, Italy
- Giovanni Luca Botto Ospedale di Garbagnate Milanese, ASST Rhodense, Rho MI, Italy
- Valeria Calvi Azienda Ospedaliero Universitaria Policlinico “G.Rodolico – San

Marco”, Catania, Italy

- Eraldo Occhetta Ospedale Maggiore, Novara, Italy
- Gemma Pelargonio Policlinico Universitario Fondazione Agostino Gemelli, Roma, Italy
- Ennio C.L. Pisanò Ospedale ‘Vito Fazzi’, Lecce, Italy
- Miguel Viscusi Ospedale “S. Anna e S. Sebastiano”, Caserta, Italy

**1.3. Endpoint Adjudication Board (EAB)**

The EAB was responsible for the review and adjudication of all potential study primary endpoints related to atrial high-rate episodes and thromboembolic cerebral events that occurred during the trial. The EAB provided standardized, systematic, and unbiased judgment of these clinical events regarding the fulfilment of the specified endpoint criteria, thus improving the quality and reliability of trial results. The roles, responsibilities, and adjudication criteria of the EAB are detailed in the Charter for endpoint adjudication version 1.0, 14-JUN-2018.

Members of the EAB:

- Andrea Campana (CHAIR) Azienda Ospedaliera Universitaria OO.RR. San Giovanni

di Dio Ruggi d'Aragona, Salerno, Italy

- Elia De Maria (MEMBER) Ospedale Ramazzini, Carpi MO, Italy
- Gennaro Miracapillo (MEMBER) Ospedale della Misericordia, Grosseto, Italy

**1.4. Statistical analysis responsibility**

Statistical analysis was performed according to the Statistical Analysis Plan version 1.0, 21-FEB-2021, by Martina Del Maestro, Daniele Giacopelli, Alessio Gargaro (Clinical Research Unit of Biotronik Italia S.p.A., Cologno Monzese MI, Italy).

Blind validation analysis was performed by Maria Vittoria Chiaruttini, Giulia Lorenzoni, Dario Gregori (the Unit of Biostatistics, University of Padua, Italy).

**1.5. Funding**

The sponsor of the study was BIOTRONIK SE & Co. KG (Woermannkehre 1, D-12359 Berlin, Germany). All costs related to the study were directly supported by the sponsor.

The sponsor ensured that all documents, information, and necessary human resources support were made available for the start and the conduct of the study (as described in the ISO14155:2020 - Clinical investigation of medical devices for human subjects - Good clinical practice).

In addition, the sponsor had the following tasks:

- Taking out the insurance of clinical trials
- Selecting suitable investigational sites and investigators in consultation with the coordinating clinical investigator
- Reporting to the authorities in accordance with national regulations

Study Project Manager was Martina Del Maestro, assisted by Clinical Project Associate Alessia Roscio (Clinical Research Unit of Biotronik Italia S.p.A., Cologno Monzese MI, Italy)

Clinical monitoring activities were conducted by Mediolanum Cardio Research S.r.l. (Milano, Italy), Biotronik Italia S.p.A. (Irene Baldassarre, Paola Napoli), Biotronik Spain S.A. (Tania Ballesteros Buitrago, Paloma Quesada), Biotronik Hungaria Kereskedelmi Kft (Gloria Szabo), Biotronik (Beijing) Medical Devices Ltd (Lan Chen), Biotronik Korea Co. Ltd (Youngsuep Kang, Ji Hwan Jung), Biotronik Asia Pacific Pte Ltd (Cindy Chan).

**1.6. Trial vigilance**

The study progression and safety were periodically reviewed by the Sponsor (BIOTRONIK SE & Co. KG), with regular biannual safety reports forwarded to the investigational sites and competent Ethics Committees. Furthermore, all Serious Adverse Events (SAEs), Serious Adverse Device Effects (SADEs), and Device Deficiencies with a SADE potential were reported by the Sponsor to the competent authorities depending on the local regulatory requirements. No unanticipated SADEs were reported during the study.

**2 Supplementary methods**

**2.1. Clinical investigation plan and amendment history**

The original clinical investigation plan (CIP, version 2.0) was released on 28-NOV-2014.

Amendment 1 was released on 10-OCT-2017 to extend the enrolment window to 120 days after implantation and to reword portions of the text for clarity.

**2.2. Study flowchart**


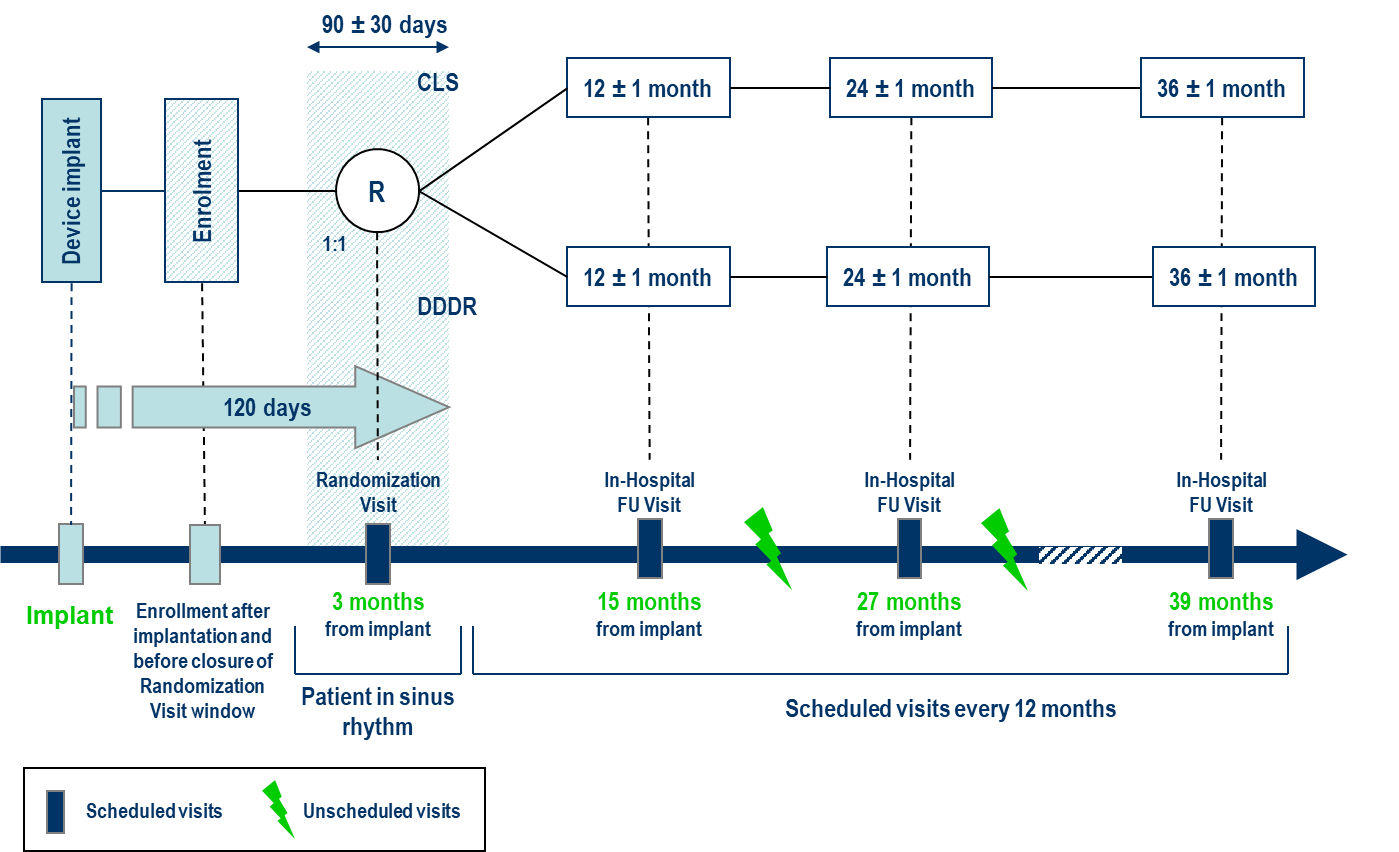


**Figure S1** Study flowchart.

**2.3. Device programming recommendations at randomization visit**

**Table S1** Device programming recommendations at randomization visit by study group

| **Parameter** | **CLS arm** | **DDDR arm** |
| --- | --- | --- |
| Mode | DDD-CLS | DDDR |
| Basic rate/night rate | 60/-- bpm | 60/-- bpm |
| Upper rate/response | 130 bpm/WBK** | 130 bpm/WBK** |
| Upper sensor rate | 120 bpm | 120 bpm |
| Rate-adaptive advanced parameters: | |  |
| Response factor | Medium | Default |
| Resting rate control | +20 bpm | --- |
| Ventricular pacing required | No* | --- |
| Mode switching | 160 bpm/DDIR | 160 bpm/DDIR |
| Onset/resolution criterion | 5/8 | 5/8 |
| 2:1 Lock-in protection***** | ON | ON |
| Atrial overdrive***** | OFF | OFF |
| Dynamic AV delay | ON | ON |
| AV hysteresis | IRS^plus^ | IRS^plus^ |
| PMT protection | ON | ON |
| AT/AF Detection (ICDs) | 190 bpm | 190 bpm |
| **Diagnostics** | **Pacemaker** | **ICD** |
| High atrial rate episodes | ON | - |
| High ventricular rate | ON** | - |
| Patient trigger | OFF | - |
| High atrial rate limit | 190 bpm | - |
| High ventricular rate limit | 180 bpm** | - |
| High ventricular rate counter | 8 events** | - |
| Thoracic impedance | ON*,** | ON*,** |
| IEGM for AT/AF | ON | Advanced ON |

***** if applicable; ****** Recommended values.

Abbreviations: AT/AF = atrial tachycardia/fibrillation; AV = atrioventricular; bpm = beats per minute; ICD = implantable cardioverter-defibrillator; IEGM = intracardiac electrogram; PMT = pacemaker mediated tachycardia; WBK = Wenckebach.

**2.4. Group sequential design**

The B3 study had a group sequential design based on the Lan-DeMets alpha spending (two-sided) function and pre-specified O’Brien-Fleming boundaries. Three-interim analyses and a final analysis were initially planned (Table S2).

**Table S2** Initial plan for group sequential study design

| **Stage** | **Boundaries** | **Error spending** | **Nominal p-value** |
| --- | --- | --- | --- |
| 1 (25%*) | (+/-) 4.3326 | 0.000015 | 0.000015 |
| 2 (50%*) | (+/-) 2.9631 | 0.003051 | 0.003045 |
| 3 (75%*) | (+/-) 2.3590 | 0.019299 | 0.018322 |
| 3 (100%*) | (+/-) 2.0141 | 0.050000 | 0.044000 |

*Proportion of primary endpoints adjudicated to test the primary study hypothesis.

**3 Supplementary results**

**3.1. Premature study termination**

On 07-FEB-2023, the EAB informed the study Project Manager that the number of expected primary endpoint events was reached, and recommended study termination according to the CIP version 2.0. In agreement with the Steering Committee, the study was terminated prematurely as of 10-FEB-2023. At that date, only the first two interim analyses had been performed. The third planned interim analysis was skipped in consequence of premature termination, to proceed with the final analysis after completion of study termination procedures, and collection and adjudication of all endpoint-related events.

The boundaries were reviewed accounting only for two interim analyses at 25% and 50% of the expected number of events and the final analysis (Table S3). The required nominal P-value for statistical significance was set to p<0.049 according to the reviewed boundaries.

Summary of performed interim analyses and final adjusted interference is provided in Table S4.

**Table S3** Group sequential study design after boundaries review

| **Stage** | **Boundaries** | **Error spending** | **Nominal p-value** |
| --- | --- | --- | --- |
| 1 (25%) | (+/-) 4.3326 | 0.000015 | 0.000015 |
| 2 (50%) | (+/-) 2.9631 | 0.003051 | 0.003045 |
| 3 (100%) | (+/-) 1.9686 | 0.050000 | 0.048999 |

**Table S4** Performed interim analyses and final adjusted inference

| **Analysis** | **CLS arm** | **DDDR arm** | **Repeated**  **95% CI for HR** | **Repeated/**  **final P-values** | **Action** |
| --- | --- | --- | --- | --- | --- |
| I interim (n=234) | 68 (57.6%) | 59 (50.9%) | 0.53-2.47 | >0.5 | Continue |
| II interim (n=466) | 129 (54.0%) | 129 (56.8%) | 0.60-1.26 | >0.5 | Continue |
| Final analysis (n=1160) | 297 (50.6%) | 319 (55.7%) | 0.72-0.99 | 0.0342 | Reject |
| Final adjusted inference* | 297 (50.6%) | 319 (55.7%) | 0.72-0.99 | 0.0350 | Reject |

*Based on the stagewise ordering of the sample space^1,2^ using the unadjusted Cox proportional hazard model for survival function stratified by investigational site (treatment as single covariate).

Abbreviations: CI = confidence interval; HR = hazard ratio.

**3.2. Endpoint adjudication**

A total of 7332 atrial high-rate episodes (AHREs) detected by implanted devices and documented by intracardiac electrogram (IEGM) recordings were adjudicated by the Endpoint Adjudication Board (EAB), along with 17 suspected cerebrovascular thromboembolic events. The results are reported in Tables S5 and S6.

**Table S5** Summary of endpoint adjudication

|  | | | **All**  **n (%)** | **Agreement**  **n (%)** | **Majority vote n (%)** |
| --- | --- | --- | --- | --- | --- |
| Total events (%) | | | 7365 | 6384 (86.7) | 981 (13.3) |
| True positive AHRE:  7236 (98.2) | Irregular forms 6871 (95.0) |  | 6871 (93.2) | 6364 (92.6) | 507 (7.4) |
|  | Regular forms 365 (5.0) | Atrial flutter | 241 (3.3) | 8 (3.3) | 233 (96.7) |
|  |  | Atrial tachycardia | 115 (1.6) | 3 (2.6) | 112 (97.3) |
|  |  | Chaotic atrial tachycardia | 9 (0.1) |  | 9 (100) |
| False positive AHRE: 96 (1.3) | | Sinus tachycardia | 3 (0.0) |  | 3 (100) |
|  |  | Electrical noise from potential  lead failure | 40 (0.5) |  | 40 (100) |
|  |  | Electromagnetic interference | 32 (0.4) |  | 32 (100) |
|  |  | Far-field oversensing | 14 (0.2) |  | 14 (100) |
|  |  | Other non-physiological signals | 7 (0.1) |  | 7 (100) |
| Thromboembolic events: 17 (0.2) | | Hemorrhagic stroke | 1 (0.0) | 1 (100) |  |
|  |  | Ischemic stroke | 11 (0.1) | 3 (27.3) | 8 (72.7) |
|  |  | Transient ischemic attack | 5 (0.1) | 5 (100) |  |
| No (AHRE) event | | | 1 (0.0) |  | 1 (100) |
| Adjudication (of AHRE) not possible | | | 15 (0.2) |  |  |

**Table S6** Duration of true positive AHREs

|  | **All AHREs**  **n (%)** | **AHRE <6 minutes**  **n (%)** | **AHRE ≥6 minutes**  **n (%)** |
| --- | --- | --- | --- |
| True positive AHRE | 7236 (100) | 2069 (28.6) | 5167 (71.4) |

**3.3. Sensitivity analyses**

The sensitivity analyses on the intention-to-treat (ITT) and per-protocol (PP) basis for the primary endpoint are summarized in Tables S7 and S8. PP analysis was done after exclusion of patients who received an incorrect programming of the pacing mode assigned by the randomization or who crossed over before the primary endpoint.

The analyses covered the following aspects:

- PP: Cox proportional hazard model for survival function stratified by investigational sites for standard errors (Table S8);
- ITT/PP: Cox proportional hazard model for survival function stratified by investigational sites and continent (Europe, Asia) for standard errors adjusted by age and sex (Tables S7/S8);
- ITT/PP: Cox proportional hazard model for survival function stratified by investigational sites for standard errors adjusted by CHA_2_DS_2_-VASc score (Tables S7/S8); and
- ITT/PP: Competitive risk regression analysis, clustering observations by investigational sites for standard errors, using treatment (CLS) as independent variable and all-cause deaths as competing risk (Tables S7/S8, Figures S2A/S2B).

The sensitivity analysis for secondary endpoints on the PP basis is shown in Table S9.

**Table S7** Sensitivity analysis for primary endpoint (intention to treat)

|  | **CLS arm**  **(n=587)** | **DDDR arm**  **(n=573)** | **Model** | **Hazard ratio**  **(95% CI)** | **P-value** |
| --- | --- | --- | --- | --- | --- |
| Primary endpoint, n (%) | 297 (50.6%) | 319 (55.7%) | Cox stratified by site adjusting by age and sex* | 0.83 (0.71–0.98) | 0.028 |
|  |  |  | Cox stratified by Europe/Asia adjusting by age and sex | 0.83 (0.71–0.97) | 0.022 |
|  |  |  | Cox stratified by site adjusting by CHA_2_DS_2_-VASc score** | 0.84 (0.71–0.99) | 0.033 |
|  |  |  | Competing risk clustering by site*** | 0.85 (0.74–0.98) | 0.028 |

*Age (years) and sex (female) hazard ratios were 1.02 (95% CI, 1.01–1.03; p<0.001) and 1.07 (95% CI, 0.90–1.26; p=0.461), respectively.

**CHA_2_DS_2_-VASc score hazard ratio was 1.05 (95% CI, 0.99–1.11; p=0.089).

***All-cause death as competing event.

**Table S8** Sensitivity analysis for primary endpoint (per protocol)

|  | **CLS arm**  **(n=532)** | **DDDR arm**  **(n=556)** | **Model** | **Hazard ratio**  **(95% CI)** | **P-value** |
| --- | --- | --- | --- | --- | --- |
| Primary endpoint, n (%) | 263 (49.4%) | 308 (55.4%) | Cox stratified by site | 0.82 (0.69–0.97) | 0.021 |
|  |  |  | Cox stratified by site adjusting by age and sex* | 0.81 (0.69–0.96) | 0.016 |
|  |  |  | Cox stratified by Europe/Asia adjusting by age and sex | 0.81 (0.69–0.95) | 0.012 |
|  |  |  | Cox stratified by site adjusting by CHA_2_DS_2_-VASc score** | 0.82 (0.69–0.97) | 0.020 |
|  |  |  | Competing risk clustering by site*** | 0.83 (0.71–0.97) | 0.019 |

*Age (years) and sex (female) hazard ratios were 1.02 (95% CI, 1.01–1.03; p<0.001) and 1.08 (95% CI, 0.91–1.29; p=0.377), respectively.

**CHA_2_DS_2_-VASc score hazard ratio was 1.06 (95% CI, 1.00–1.13; p=0.047).

***All-cause death as competing event.

**Table S9** Sensitivity analysis for secondary endpoints (per protocol)

| **Events** | **CLS arm**  **(532 patients)** | **DDDR arm**  **(556 patients)** | **Hazard ratio (95% CI)** | **P-value** |
| --- | --- | --- | --- | --- |
| AHRE, n (%) |  |  |  |  |
| ≥6 minutes | 259 (48.8) | 305 (54.9) | 0.82 (0.69-0.97) | **0.019** |
| ≥7 days | 55 (9.4) | 46 (8.0) | 1.21 (0.81–1.81) | 0.36 |
| Permanent AF | 41 (7.7) | 46 (8.3) | 0.96 (0.63-1.48) | 0.86 |
| Stroke or TIA | 10 (1.9) | 5 (0.9) | 2.18 (0.73-6.46) | 0.16 |
| Hospitalization for WHF | 14 (2.6) | 9 (1.6) | 1.66 (0.72-3.87) | 0.24 |
| All-cause death | 36 (6.7) | 40 (7.2) | 0.88 (0.55-1.40) | 0.58 |

Data are n (% of patients) unless otherwise stated.

Abbreviations: AF = atrial fibrillation; AHRE = atrial high-rate episode; CI = confidence interval; TIA = transient ischemic attack; WHF = worsening heart failure.

**Figure S2** Plots of cumulative incidence by study groups based on competing-risk regressions reported in Tables S7 and S8 (the last rows) for the intention-to treat (A) and per-protocol (B) analyses.

**A.** **B.**

 ****

**4 Supplementary discussion**

**4.1 Closed Loop Stimulation functioning principle**

Closed Loop Stimulation (CLS) is a proprietary rate-adaptive pacing system described for the first time in the early 1990s.^3,4^ It is based on monitoring impedance trends during systoles. For each sensed or paced event, the trains of high-frequency, subthreshold, unipolar current pulses are delivered from the right ventricular lead tip during systolic blood ejection. The main contribution to the voltage drop (and hence impedance) is provided by a small volume surrounding the lead tip and containing a varying amount of blood and myocardial tissue. During systolic ejection, the progressively reduced amount of blood leads to an increase in the impedance trend (Figure S3), which has been shown to be associated with the right ventricular dP/dtmax,^5^ a well-known surrogate for contraction speed and contractility. Fluctuations in contractility are reflected in corresponding variations in impedance trends detected by the CLS algorithm during systole. CLS reacts by adjusting the pacing rate according to the variation detected.

CLS is therefore integrated into the autonomic regulation of cardiac output, which explains the origin of the name “closed loop”: an increase in heart rate determined by CLS (in response to a modification in contractility) affects cardiac output and triggers intrinsic regulation mechanisms, including contractility, which is in turn detected by CLS, thus “closing the loop”. CLS has been shown to detect and react not only to physical activity, but also to active standing, handgrip, cold pressor test, mental stress, and dobutamine infusion.^6-11^ Such a “closed loop” mechanism is absent in rate-adaptive systems driven by accelerometer sensors, which can only react to body movements.


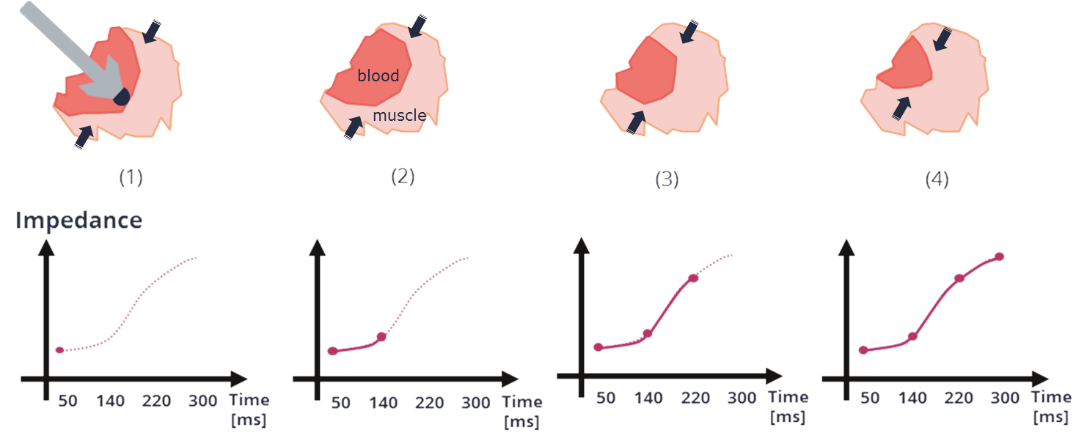


**Figure S3**. Increase in the right ventricular impedance trend during systole. Impedance is monitored in a temporal window from 50 ms to 300 ms after each sensed or paced ventricular event. Due to varying amount of blood and myocardial tissue during ejection, impedance is expected to increase. The increase rate is associated with the right ventricular dP/dtmax, a surrogate for contraction speed and contractility.

**4.2** **Review of previous studies on CLS in sinus node disease**

Three smaller studies with a shorter follow-up (6–12 months) and non-adjudicated or partly adjudicated AHRE data have previously reported the benefit of CLS in reducing AHRE burden compared to alternative pacing strategies. In the BURDEN I study, Puglisi et al.^12^ randomized 149 patients with brady-tachy syndrome to accelerometer-based DDDR pacing, atrial overdrive pacing (DDD+ algorithm), or CLS, and followed them for 6 months. Calculated as the cumulative duration of periods with atrial rate ≥150 bpm, atrial tachyarrhythmia burden was significantly lower in the CLS group (20 ± 63 minutes per day, P<0.01) than in the DDDR (56 ± 184 min/day) or DDD+ group (63 ± 114 min/day).^12^

In the subsequent BURDEN II study, 451 patients with brady-tachy syndrome, severe bradycardia, and a documented episode of atrial fibrillation (AF) were randomized to accelerometer-based DDDR pacing, atrial overdrive pacing (DDDR+ algorithm), or CLS, and were followed for 9 months with pre-specified mode crossovers every 3 months.^13^ Atrial tachyarrhythmia burden was calculated as the cumulative duration of periods with atrial rate ≥180 bpm, adjusted for false-positive episodes and reported as geometric means after using a simple log transformation to handle large and skew burden distributions. The burden was signiﬁcantly lower in the CLS group (0.04%, p<0.001) than in the DDDR (0.08%) or DDDR+ group (0.12%).^13^

Ikeda et al.^14^ retrospectively evaluated atrial tachyarrhythmia burden in 146 patients with sinus node disease (SND) and AF history, and found a significantly lower median burden at 12 months for CLS (0% [interquartile range, 0%–2.5%]) than for fixed-rate pacing (1.6% [0%–11.0%]; p<0.01) or for DDDR pacing using an accelerometer sensor or a combination of accelerometer and minute ventilation sensors (1.0% [0.1%–9.3%]; p=0.04). In a logistic regression analysis, the risk of atrial tachyarrhythmia occurrence was lower in CLS even after adjusting for potential confounders (odds ratio, 0.31; 95% confidence interval, 0.12–0.80; p=0.02).

Our large, randomized B3 trial with fully adjudicated AHRE data demonstrated the long-term benefit of CLS in time-to-event analysis in patients with SND who predominantly did not have known AF (63.8%). Although we observed no benefit in the subset of patients with AF history, all three previous studies reported a significant reduction in total atrial tachyarrhythmia burden with CLS despite AF history in most^12^ or all^13,14^ patients. The strict adjudication process for AHREs in our study (see Study limitations in the main article) did not allow us to assess total AHRE burden and verify these previous findings. However, taking into account all four studies, it is plausible that CLS reduces the total arrhythmia burden although it does not postpone the first device-detected AHRE in patients with AF history.

**5 References**

1. Wassmer G, Brannath W. Chapter 4. Confidence Intervals, p-Values, and Point Estimation. In:

Group Sequential and Confirmatory Adaptive Designs in Clinical Trials. 1st ed. Springer Series

in Pharmaceutical Statistics. Springer Cham; 2016:83-100. <https://doi.org/10.1007/978-3->

319-32562-0

2. Wassmer G, Pahlke F. Rpact: Confirmatory Adaptive Clinical Trial Design and Analysis.; 2023. <https://www.rpact.org>, <https://www.rpact.com>, <https://github.com/rpact-com/rpact>,

https://rpactcom. github.io/rpact/

3. Schaldach M, Hutten H. Intracardiac impedance to determine sympathetic activity in rate responsive pacing. Pacing Clin Electrophysiol 1992;15(11 Pt 2):1778-1786.

4. Schaldach M, Ebner E, Hutten H, von Knorre GH, Niederlag W, Rentsch W, Volkmann H, Weber D, Wunderlich E. Right ventricular conductance to establish closed-loop pacing. Eur Heart J 1992;13(Suppl. E):104-112.

5. Osswald S, Cron T, Graedel C, Hilti P, Lippert M, Stroebel J, Schaldach M, Buser P, Pfisterer M. Closed-loop stimulation using intracardiac impedance as a sensor principle: correlation of right ventricular dP/dtmax and intracardiac impedance during dobutamine stress test. Pacing Clin Electrophysiol 2000;23:1502-1508.

6. Binggeli C, Duru F, Corti R, Sudano I, Spieker LE, Turina A, Westermann P, Ostermeier M, Rahn M, Luescher TF, Noll G, Candinas R. Autonomic nervous system-controlled cardiac pacing: a comparison between intracardiac impedance signal and muscle sympathetic nerve activity. Pacing Clin Electrophysiol 2000;23:1632-1637.

7. Griesbach L, Gestrich B, Wojciechowski D, Weyers G, Toenges J, Schier M, Danilovic D. Clinical performance of automatic closed loop stimulation systems. Pacing Clin Electrophysiol 2003;26:1432-1437.

8. Santini M, Ricci R, Pignalberi C, Biancalana G, Censi F, Calcagnini G, Bartolini P, Barbaro V. Effect of autonomic stressors on rate control in pacemakers using ventricular impedance signal. Pacing Clin Electrophysiol 2004;27:24-32.

9. Chandiramani S, Cohorn LC, Chandiramani S. Heart rate changes during acute mental stress with Closed Loop Stimulation: report on two single-blinded, pacemaker studies. Pacing Clin Electrophysiol 2007;30:976-984.

10. Coenen M, Malinowski K, Spitzer W, Schuchert A, Schmitz D, Anelli-Monti M, Maier SK, Estlinbaum W, Bauer A, Muehling H, Kalscheur F, Puerner K, Boergel J, Osswald S. Closed loop stimulation and accelerometer-based rate adaptation: results of the PROVIDE study. Europace 2008;10:327-333.

11. Quaglione R, Calcagnini G, Censi F, Piccirilli F, Iannucci L, Raveggi M, Biancalana G, Bartolini P. Autonomic function during closed loop stimulation and fixed rate pacing: heart rate variability analysis from 24-hour Holter recordings. Pacing Clin Electrophysiol 2010;33:337-342.

12. Puglisi A, Altamura G, Capestro F, Castaldi B, Critelli G, Favale S, Pavia L, Pettinati G. Impact of closed-loop stimulation, overdrive pacing, DDDR pacing mode on atrial tachyarrhythmia burden in brady-tachy syndrome. A randomized study. Eur Heart J 2003;24:1952-1961.

13. Puglisi A, Favale S, Scipione P, Melissano D, Pavia L, Ascani F, Elia M, Scaccia A, Sagone A, Castaldi B, Musacchio E, Botto GL. Overdrive versus conventional or closed-loop rate modulation pacing in the prevention of atrial tachyarrhythmias in Brady-Tachy syndrome: on

behalf of the Burden II Study Group. Pacing Clin Electrophysiol 2008;31:1443-1455.

14. Ikeda S, Nogami A, Inoue K, Kowase S, Kurosaki K, Mukai Y, Tsutsui H. Closed-loop stimulation

as a physiological rate-modulated pacing approach based on intracardiac impedance to lower

the atrial tachyarrhythmia burden in patients with sinus node dysfunction and atrial fibrillation. J Cardiovasc Electrophysiol 2020;31:1187-1194.
